# Photorepair of Either CPD or 6-4PP DNA Lesions in Basal Keratinocytes Attenuates Ultraviolet-Induced Skin Effects in Nucleotide Excision Repair Deficient Mice

**DOI:** 10.3389/fimmu.2022.800606

**Published:** 2022-03-29

**Authors:** Gustavo S. Kajitani, Carolina Quayle, Camila C. M. Garcia, Wesley L. Fotoran, Juliana F. R. dos Santos, Gijsbertus T. J. van der Horst, Jan H. J. Hoeijmakers, Carlos F. M. Menck

**Affiliations:** ^1^ Departamento de Microbiologia, Instituto de Ciências Biomédicas, Universidade de São Paulo, São Paulo, Brazil; ^2^ Departamento de Ciências Biológicas (DECBI), Instituto de Ciências Exatas e Biológicas, Universidade Federal de Ouro Preto, Ouro Preto, Brazil; ^3^ Departamento de Parasitologia, Instituto de Ciências Biomédicas, Universidade de São Paulo, São Paulo, Brazil; ^4^ Department of Molecular Genetics, Erasmus University Medical Center, Rotterdam, Netherlands; ^5^ University Hospital of Cologne, Cologne Excellence Cluster for Cellular Stress Responses in Aging-Associated Diseases (CECAD), Institute for Genome Stability in Aging and Disease, Cologne, Germany; ^6^ Princess Maxima Center for Pediatric Oncology, ONCODE Institute, Utrecht, Netherlands

**Keywords:** photolesions, photolyase, nucleotide excision repair, xeroderma pigmentosum, UVB ultraviolet radiation, inflammation, cell death

## Abstract

Ultraviolet (UV) radiation is one of the most genotoxic, universal agents present in the environment. UVB (280-315 nm) radiation directly damages DNA, producing cyclobutane pyrimidine dimers (CPDs) and pyrimidine 6-4 pyrimidone photoproducts (6-4PPs). These photolesions interfere with essential cellular processes by blocking transcription and replication polymerases, and may induce skin inflammation, hyperplasia and cell death eventually contributing to skin aging, effects mediated mainly by keratinocytes. Additionally, these lesions may also induce mutations and thereby cause skin cancer. Photolesions are repaired by the Nucleotide Excision Repair (NER) pathway, responsible for repairing bulky DNA lesions. Both types of photolesions can also be repaired by distinct (CPD- or 6-4PP-) photolyases, enzymes that specifically repair their respective photolesion by directly splitting each dimer through a light-dependent process termed photoreactivation. However, as photolyases are absent in placental mammals, these organisms depend solely on NER for the repair of DNA UV lesions. However, the individual contribution of each UV dimer in the skin effects, as well as the role of keratinocytes has remained elusive. In this study, we show that in NER-deficient mice, the transgenic expression and photorepair of CPD-photolyase in basal keratinocytes completely inhibited UVB-induced epidermal thickness and cell proliferation. On the other hand, photorepair by 6-4PP-photolyase in keratinocytes reduced but did not abrogate these UV-induced effects. The photolyase mediated removal of either CPDs or 6-4PPs from basal keratinocytes in the skin also reduced UVB-induced apoptosis, ICAM-1 expression, and myeloperoxidase activation. These findings indicate that, in NER-deficient rodents, both types of photolesions have causal roles in UVB-induced epidermal cell proliferation, hyperplasia, cell death and inflammation. Furthermore, these findings also support the notion that basal keratinocytes, instead of other skin cells, are the major cellular mediators of these UVB-induced effects.

## Introduction

Ultraviolet (UV) radiation is the main exogenous physical factor involved in carcinogenesis, capable of directly damaging DNA by inducing the formation of covalent bonds between adjacent pyrimidines of the same DNA strand, producing pyrimidine dimers (1). The main DNA photolesions caused by UV radiation are cyclobutane pyrimidine dimers (CPDs) and pyrimidine (6–4) pyrimidone photoproducts (6-4PPs). These lesions distort the helical DNA duplex molecule by interfering with proper base-pairing and thus interfere with essential cellular processes, such as transcription and replication (2, 3). Both DNA photolesions can be repaired by photolyases, enzymes capable of directly repairing either CPDs or 6-4PPs through a process known as photoreactivation. During this photorepair process, the photolyase binds to the pyrimidine dimer and breaks the covalent bond in a light-dependent reaction, reverting the lesion back to the original monomers (4). Moreover, photolyases act in a specific manner, with CPD-photolyases repairing only CPDs and 6-4PP-photolyases repairing only 6-4PPs. Due to their specificities, photolyases can be used as tools to study the distinct effect of each photolesion (5).

Although photolyase genes are generally found in all domains of life, they are absent in some groups, most notably placental mammals (6). Mammals remove CPD and 6-4PP lesions by the nucleotide excision repair (NER) pathway. NER is a well-conserved mechanism responsible for removing a wide variety of lesions that distort the DNA double helix structure, including those induced by the UV component of sunlight (7). Two distinct NER sub-pathways differ in DNA damage recognition: transcription-coupled repair (TC-NER) and global genome repair (GG-NER). DNA lesions at the transcribed strand of active genes stall RNA polymerase II transcription, signaling for TC-NER. DNA damage located throughout the genome are recognized by the XPC/HR23B protein complex for GG-NER (8). As a result of these distinct mechanisms, TC-NER and GG-NER differ in their capacity to recognize DNA lesions. Particularly in mice, CPDs are essentially removed mainly by TC-NER, which rescues transcription and thus promotes cellular survival, as CPDs are poorly repaired in the non-transcribed strand and non-transcribed genomic regions. In contrast, 6-4PPs are rapidly repaired in the entire genome by GG-NER (9).

Xeroderma Pigmentosum (XP), a rare recessive, autosomal genetic disorder, is mainly caused by mutations in genes (*XPA-XPG*) involved in the NER pathway (10). A milder type of XP that does not present defective NER is named XP variant (XP-V). XP-V is instead caused by mutations in the *POLH* gene, which codes for the translesion DNA polymerase eta (8). XP is characterized primarily by a marked increased risk of skin neoplasia and cutaneous hypersensitivity to UV radiation, with XP patients often displaying severe sunburn and blistering of the skin after minimal sunlight exposure (8, 11).

UV radiation can promote cutaneous inflammation, in which skin cells, especially keratinocytes, produce and activate proteins associated with pro-inflammatory processes. These include the transcription factor NF-kB, cytokines such as IL-1α, IL-1β, TNFα, as well as proteins involved in the inflammasome complex (12–14). In addition, these molecules contribute to the expression of proteins integral to the inflammatory process, such as ICAM-1 (15) and metalloproteinases (MMPs) that allow neutrophils and macrophages to enter the skin tissue and initiate inflammation.

Interestingly, the transgenic expression of CPD-photolyase and photorepair in NER-proficient mice reduces UV-induced skin inflammation, suggesting that DNA damage itself is sufficient to trigger this biological process (16). Photorepair of CPD, but not 6-4PP, in NER-proficient mice also inhibits other UV-induced effects, namely skin hyperplasia, cell death and tumorigenesis (17). Photorepair of CPD, but not 6-4PP, in cell cultures also reduces UV-induced cell death in NER-proficient cells. However, in cells derived from XP patients, photorepair by either CPD or 6-4PP-photolyase reduces the apoptotic effect of UV radiation. Concomitant photorepair by both photolyases further reduced UV-induced apoptosis, indicating that both CPD and 6-4PP lesions participate in UV-induced effects in NER-deficient models (2).

In this study, we show that *in vivo* photoreactivation through K-14 promoter driven expression of either CPD or 6-4PP-photolyase in basal keratinocytes reduces acute UVB-induced apoptosis in NER-deficient *Xpa* knockout mice. The photoremoval of either photolesion in this model also decreased UV-induced inflammation, as the expression of either photolyase diminished ICAM-1 levels and active neutrophils present in the skin of UVB-irradiated *Xpa*-deficient mice. In contrast, only CPD removal abolished chronic UV-induced skin cell proliferation and hyperplasia, with 6-4PP removal having a minor impact on these UV-induced effects. These findings indicate that both types of DNA lesions directly participate in inducing apoptosis, inflammation, and hyperplasia. Furthermore, these results also support the notion that basal keratinocytes are the key mediators of these UV-induced effects.

## Materials and Methods

### Mouse Lines

*Xpa* knockout mice expressing CPD- or 6-4PP-photolyase were obtained by generational crossing between the *Xpa^-/-^* mice described in (18) with transgenic K14-CPD-PL or K-14-64PP-PL photolyase mice (19), hereafter referred to as CPD or 6-4PP-photolyase mice, respectively. Both photolyase genes are under the control of the basal keratinocyte-specific K-14 promoter (19). All strains used in this project were initially obtained at the Erasmus Medical Center University, Rotterdam (the Netherlands) were in a C57BL/6J or hairless C57BL/6J/SKH-1 genetic background, established models for UV radiation (18, 20). *Xpa^-/-^* mice were maintained by homozygous crosses, while photolyase expressing genes and the *hairless* gene were maintained by heterozygous crosses. All animals used for experiments were 8- to 10-week-old. As there are no differences regarding UV sensitivity between males and females, mice of both genders were used for all experiments. Housing, breeding, genotyping, and experimentation were performed in accordance with the regulations established by the ethical committee for experimentation with animals of the Institute of Biomedical Sciences of the University of Sao Paulo (Protocol #121-11-03).

Genotyping was performed on mouse tail DNA followed by polymerase chain reaction (PCR) of target genes. PCR conditions of the K14-*CPD-PL* and K14-*64PP-PL* genes are described in **Supplementary Tables S1, S2**, and primer sequences in **Supplementary Table S3**.

### UV Radiation and Photoreactivation of Mice

Mice were irradiated with a Philips TL12-40W UVB lamp, using a VLX-3W UV dosimeter (Vilber Loumart, Torcy, France) to measure the intensity of UV radiation. No UVC (254 nm) radiation was detected, and UVA (365 nm) radiation was below <0.05 J/m^2^/s. The distance between the UVB lamp and the mice dorsal skin was 1.10 meters. Immediately after UV irradiation, mice were exposed to photoreactivating light (four white lamps Polylux XL F36W/840) for three h, positioned 40 cm above the mice. Minimal erythemal dose (MED) of *Xpa^-/-^* mice was determined as 20 J/m^2^ of UVB by analyzing the macroscopic induction of erythema, wounding, skin peeling, skin thickening and pigmentation.

### Chronic UV Irradiation of Hairless *Xpa^-/-^* Mice for Assessment of Tissue Hyperplasia and Cell Proliferation

*Xpa^-/-^* hairless mice expressing either CPD- or 6-4PP-photolyase were irradiated for 30 consecutive days, at approximately 2:00 pm, with a 1 MED UVB (20 J/m^2^) dose followed by 3 h photoreactivation. Animals were observed daily for signs of distress, epidermal thickness, and pigmentation. 48 h after the last day of irradiation, mice (n=4) were euthanized and 1 cm^2^ mice dorsal skin was collected. Two h prior to euthanasia, animals were intra-peritoneally injected with BrdU (5 mg) for cell proliferation analysis.

### Tissue Fixation for Histology Analysis

Skin samples were fixed overnight at 4°C in 4% formaldehyde (Merck, Kenilworth, NJ, USA). Samples were then dehydrated at room temperature by sequential immersion for 1 h in each of the following solutions: PBS 1X, 50% ethanol (Merck), 70% ethanol, 80% ethanol, 90% ethanol, 2x 100% ethanol and 2x xylene (Sigma-Aldrich, Saint Louis, MO, USA). After dehydration, samples were twice incubated in paraffin 60°C for 1 h each, mounted in paraffin blocks and kept at RT until further processing. Skin tissue sections (5 μm) were obtained using a microtome, placed on Starfrost (Knittel-Glaser) slides and kept in 10% ethanol at 50°C until total fluid evaporation. For fixation on the slide, skin sections were maintained at 37°C overnight and stored at RT until staining.

### Quantification of Epidermal Thickness

Tissue slides were deparaffinized and hydrated through sequential immersion in xylene (100% twice), ethanol (100% twice, 95%, 70% and 50%) and dH_2_O under room temperature. Slides were then stained with hematoxylin and eosin (H&E). Stain excess was washed under indirect water flow, and tissue was subsequently dehydrated through immersion in ethanol and xylene. Slides were then mounted using Entellan and Menzel-Glass coverslips.

Epidermal thickness was quantified using Axiovert 200 (Zeiss, Oberkochen, Germany) optical microscope under a 100x objective. Epidermal thickness was defined as the distance between the end of the outer epidermal layer and the basal lamina. Invagination sites, such as sweat glands and hair follicles were not considered in this analysis. Three measurements were performed per field, using three fields in each slice, and three slices per animal, with a total of twenty-seven measurements per animal. Axiovision Rel. 4.8 (Zeiss) software was used for quantification.

### Tissue Cell Proliferation

BrdU detection using immunohistochemistry was performed to quantify cell proliferation. Tissue slides were deparaffinized and hydrated as previously described, then incubated for 30 min in 50% methanol 1% H_2_O_2_ (30%, Merck) for endogenous peroxidase inactivation, followed by two PBS washes. Samples were then incubated in pepsin (18 U/ml) diluted in 100 mM HCl at 37°C for 30 min, followed by two PBS washes and incubation at 56°C for 20 min in 1 M HCl. pH was neutralized with 100 mM sodium borate in PBS (pH 8.5), followed by three PBS washes. Slides were incubated in blocking solution (5% FBS in 1% PBS/BSA) for 10 min, at RT, followed by incubation with anti-BrdU (M0744, DAKO), diluted 1:100 in blocking solution overnight at 4°C. Slides were washed in PBS and incubated for 1 h with HRP anti-mouse (Sigma-Aldrich, A9044), diluted 1:100 in blocking solution. Substrate reaction was done with 3,3’-Diaminobenzidine (DAB, Spring) until nuclei were stained. Counter staining was performed with hematoxylin (Merck). Slides were mounted with Entellan and coverslips. Images were obtained with Axiovert 200 Optic Microscope (Zeiss) under 100x objective using Axiovision Rel. 4.8 (Zeiss) software. We performed three blind measurements per skin tissue of BrdU^+^ basal and suprabasal cells, analyzing 3 slices per animal. Quantification of BrdU-positive cells was performed by calculating the ratio between stained basal layer cells and total basal layer cells, while quantification of suprabasal BrdU-positive cells was performed considering the ratio between these cells and the total number of basal layer cells.

### Immunohistochemistry for the Detection of Photolesions

CPD and 6-4PP were immunodetected in skin tissue sections to confirm that the photolyases expressed in the mice models were repairing their respective photolesions. Tissue slides were deparaffinized and hydrated as previously described, then incubated in 18 U/ml pepsin diluted in 100 mM HCl at 37°C for 30 min. Slides were washed twice using PBS, then incubated at 56°C for 20 min in 1 M HCl, followed by three PBS washes. Tissues were then incubated for 20 min in blocking solution (5% FBS in 1% PBS/BSA) at RT. After blocking, tissues were incubated overnight at 4°C in anti-CPD (TDM-2, Cosmo Bio, Tokyo, Japan) or anti-6-4PP (64M-2, Cosmo Bio), diluted 1:1000 and 1:300, respectively, in the blocking solution. Slides were then washed twice with PBS and incubated in secondary antibody anti-mouse IgG conjugated with Alexa fluor 555 for 90 min. Slides were washed twice with PBS, followed by counterstaining with DAPI fluoroshield (Sigma-Aldrich) solution. Images were obtained with Axiovert 200 Optic Microscope (Zeiss) under 40x objective using Axiovision Rel. 4.8 (Zeiss) software.

### Acute Irradiation of *Xpa^-/-^* Mice for *In Vivo* Assessment of Inflammation and Cell Death

Photolyase-expressing *Xpa^-/-^* mice were anesthetized and shaved 24 h before irradiation. Mice were then irradiated with a single UV-dose of 200 J/m^2^ (10 MED).

To assess early inflammation induced by UVB light, mice (n=4) were injected with anti-ICAM-1/DiD fluorophore (excitation 640 nm, emission 680 nm) nanoparticles to detect ICAM-1 expression 6 h after UVB irradiation (21). Furthermore, after 6 and 24 h of irradiation, mice were inoculated with the XenoLight RediJect Chemiluminescent Inflammation Probe (PerkinElmer) to detect active, myeloperoxidase-expressing neutrophils (n=2). Probe fluorescence and chemiluminescence were detected using the *in vivo* imaging system (IVIS) Spectrum (PerkinElmer), located in the Core Facility Center for Research Support of the University of Sao Paulo (CEFAP-USP). We also used the MMPSense 645 FAST probe (PerkinElmer) to detect several MMPs (2, 3, 7, 9, 12, and 13, n=2).

Mice were assessed for *in vivo* cell death (n=3) 48 h after UV irradiation using Annexin-V/DiD fluorophore nanoparticles (22), injected intravenously 2 h prior to detection by IVIS Spectrum.

When imaging, mice were kept under anesthesia using isoflurane. Image analysis and quantification was performed with Living Image 4.0 (PerkinElmer) software. Radiances were normalized using non-irradiated control mice.

### Statistical Analysis

Data were expressed as mean ± standard deviation and analyzed with one-way ANOVA followed by Bonferroni’s multiple comparison test. p value < 0.05 was considered significant, with “*” indicating p ≤ 0.05, “**” p ≤ 0.01 and “***” p ≤ 0.001.

## Results

### Effects of Basal Keratinocyte-Specific Photoremoval of CPD or 6-4PP on Hyperplasia and Cell Proliferation in Chronically Irradiated *Xpa-/-* Mice

**Figure 1:**
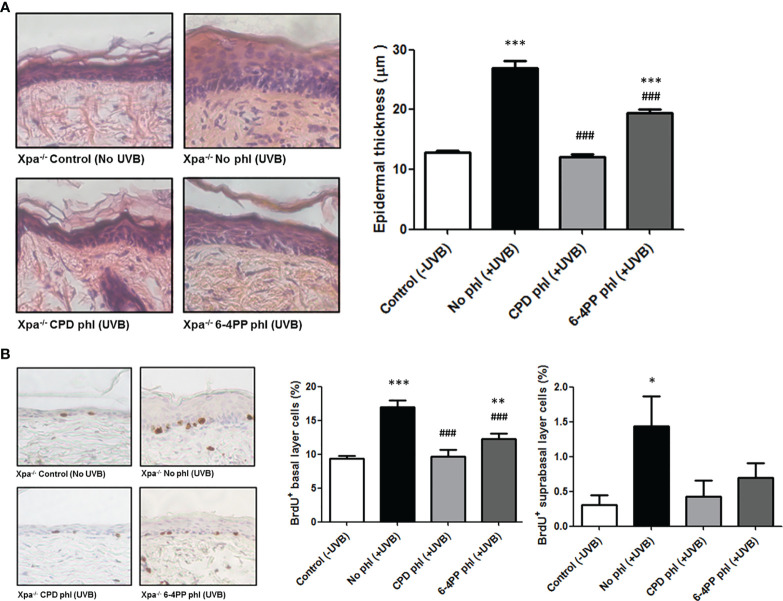
CPD removal in keratinocytes abrogates UV-induced hyperplasia and cell proliferation, while 6-4PP removal decreases these effects in Xpa-/- mice. **(A)** Epidermal thickness of Xpa-/- mice daily irradiated or not with UVB (20 J/m2) followed by photoreactivation of CPD- or 6-4PP-Photolyase (phl) for 30 consecutive days. Quantitative analysis of epidermal thickness was performed by perpendicular measurements of the tissue extension in skin sections stained with H&E (n = 4), with representative images (40x) shown. **(B)** Quantification of cell proliferation in the basal and suprabasal layers of chronically UV-irradiated Xpa-/- mice, with representative images (40x). Tissues were stained for BrdU+ cells by immunohistochemistry counterstained with hematoxylin (n = 4). Asterisks (*) indicate a statistically significant difference between the designated group and the negative, Xpa-/- non-irradiated, controls, while the pound signs (#) indicate a significant difference between the designated group and the positive control group, Xpa-/- mice with no photolyase UVB-irradiated. “*” or “#”: p<0.05, “**” or “##”: p<0.01, and “***” or “###”: p<0.001.

NER-deficient *Xpa* ^-/-^ mice show hypersensitivity to UV radiation, as low UVB doses can produce excessive skin abrasion (19). Thus, establishing a Minimal Erythemal Dose (MED) was necessary to determine a biologically relevant dose for the experiments. The MED for *Xpa^-/-^* hairless mice was determined to be 20 J/m^2^, used as a reference dose henceforth. To assess the role of CPDs and 6-4PPs on the induction of hyperplasia, mice (n=4) received over 30 days a single daily dose of 20 J/m^2^ UVB radiation followed by 3 h exposure to photoreactivating light to allow photorerepair by the respective photolyase, an exposure time previously shown to remove both photolesions by transgenic photolyase expression in basal keratinocytes (19), (**Supplementary Figure S1**). Epidermal thickness was quantified using H&E-stained sections (**Figure 1A**). *Xpa^-/-^* mice not expressing any photolyase under chronic UV radiation developed hyperplasia, while CPD photorepair in keratinocytes inhibited this UV-induced effect, evidencing the causative role of CPD lesions for UVB-induced hyperplasia. Curiously, removing 6-4PPs in keratinocytes also affected UVB-induced hyperplasia, decreasing but not abrogating this chronic UV response in *Xpa^-/-^* mice.

UVB-induced cell proliferation was also analyzed in these chronically irradiated mice (**Figure 1B**). CPD photoremoval in keratinocytes again prevented the UVB-induced cell proliferation effect both in basal and suprabasal epidermal layers. Similarly, the removal of 6-4PPs attenuated this effect in the basal layer and fully inhibited it in the suprabasal epidermal layer of *Xpa^-/-^* mice, corroborating the hyperplasia results.

### Photorepair of Either CPDs or 6-4PPs in Basal Keratinocytes Reduces UV-Induced Apoptosis in *Xpa^-/-^* Mice

Apoptotic cell death was analyzed *in vivo* through nanoparticle probes linked to Annexin-V and DiD-fluorophore (**Supplementary Figure S2**). In addition, higher UVB dose (200 J/m^2^, or 10 MED) was used to evaluate the acute, UV-induced *in vivo* effects.

**Figure 2:**
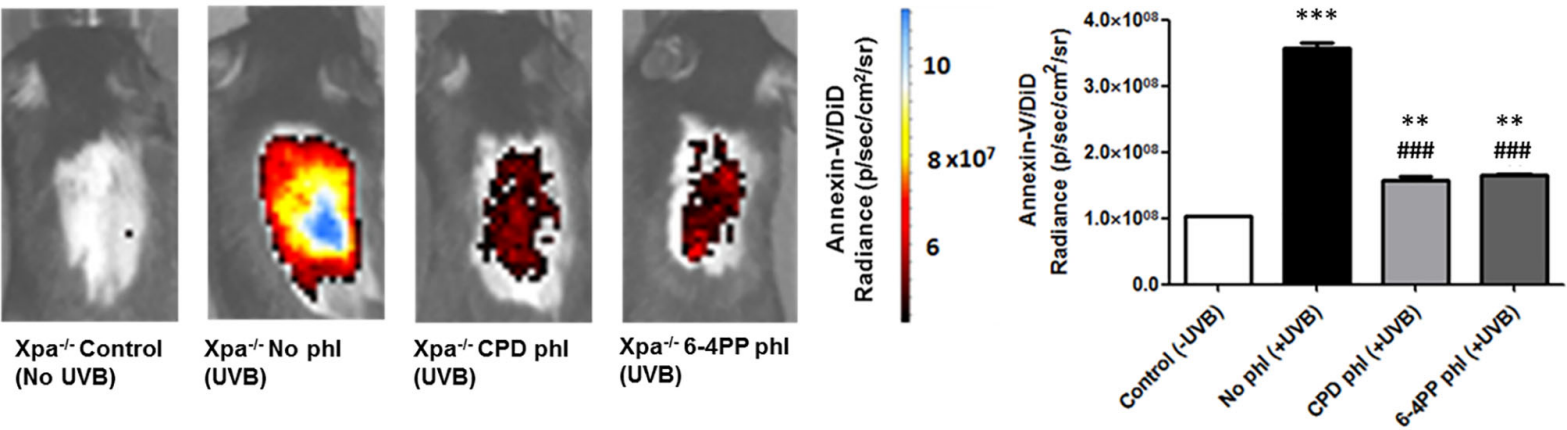
*In vivo* effect of keratinocyte-specific photorepair of CPDs and 6-4PPs on UVB-induced apoptosis in Xpa-/- mice. Apoptosis was analyzed by *in vivo* imaging using Annexin-V/DiD fluorophore containing nanoparticles, 48 h after UVB (200 J/m2) irradiation. Radiance of DiD containing nanoparticles were quantified in the central region of Xpa-/- mice exposed dorsal skin using Living Image 4.0 software, n = 3. Asterisks (*) indicate a statistically significant difference between the designated group and the negative, Xpa-/- non-irradiated, controls, while the pound signs (#) indicate a significant difference between the designated group and the positive control group, Xpa-/- mice with no photolyase UVB-irradiated. “*” or “#”: p<0.05, “**” or “##”: p<0.01, and “***” or “###”: p<0.001.

Results demonstrated that 48 h after UVB irradiation immediately followed by photoreactivation, expression of CPD-photolyase in keratinocytes of *Xpa^-/-^* mice significantly reduced the apoptotic signal compared to *Xpa^-/-^* mice not expressing photolyases, suggesting the participation of persistent CPDs in UVB-induced *in vivo* cell death (n=3). Furthermore, similar results were obtained with 6-4PP-photolyase-expressing mice, suggesting that both CPDs and 6-4PPs participate in apoptosis triggering events following UV irradiation in these NER-deficient mice (**Figure 2**).

### UVB-Induced Inflammation in *Xpa^-/-^* Mice Is Reduced by CPD or 6-4PP-Photorepair in Basal Keratinocytes

**Figure 3:**
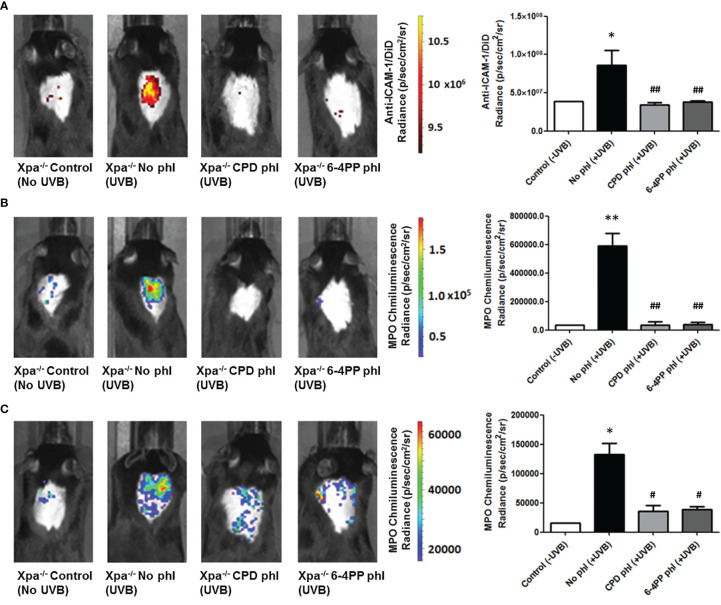
Leukocyte infiltration and activation in vivo imaging of Xpa-/- mice expressing CPD or 6-4PP-photolyase in basal keratinocytes. **(A)** *In vivo* imaging of ICAM-1, an inflammation marker, coupled to a DiD fluorophore containing nanoparticle. Imaging was performed in photolyase (CPD- or 6-4PP-phl) expressing Xpa-/- mice 6 h after irradiation with 200 J/m2 UVB, n = 4. **(B, C)** MPO, an active neutrophil marker, was measured *in vivo* 6 **(B)** and 24 h **(C)** after UVB radiation of Xpa-/- mice by using a chemiluminescent probe, n = 2. Radiance and luminescence were measured using Living Image 4.0 software. Asterisks (*) indicate a statistically significant difference between the designated group and the negative, Xpa-/- non-irradiated, controls, while the pound signs (#) indicate a significant difference between the designated group and the positive control group, Xpa-/- mice with no photolyase UVB-irradiated. “*” or “#”: p<0.05, “**” or “##”: p<0.01.

UVB-induced inflammation was measured 6 and 24 h after UVB (200 J/m^2^) irradiation and photoreactivation. Using *in vivo* probes (**Supplementary Figure S3**), we measured the expression ICAM-1, a cell surface protein responsible for neutrophil adhesion. We also used a commercial chemiluminescent probe for detecting myeloperoxidase (MPO), an enzyme highly expressed by active neutrophils and a key mediator of inflammation-dependent oxidative stress. Interestingly, both CPD or 6-4PP removal in keratinocytes decreased ICAM-1 levels (n=4) 6 h after UVB irradiation (**Figure 3A**). Similarly, CPD and 6-4PP photoremoval also lessened the infiltration of active neutrophils in the skin, as measured by *in vivo* MPO expression 6 and 24 h (n=2) after UVB irradiation (**Figures 3B, C** respectively), as well as shown in H&E-stained skin sections (**Supplementary Figure S4**), which indicates that these two photolesions participate in the inflammatory event of leukocyte tissue extravasation following UV irradiation.

### Neither CPD nor 6-4PP Photoremoval Altered MMP Activation in UVB Irradiated *Xpa^-/-^* Mice

**Figure 4:**
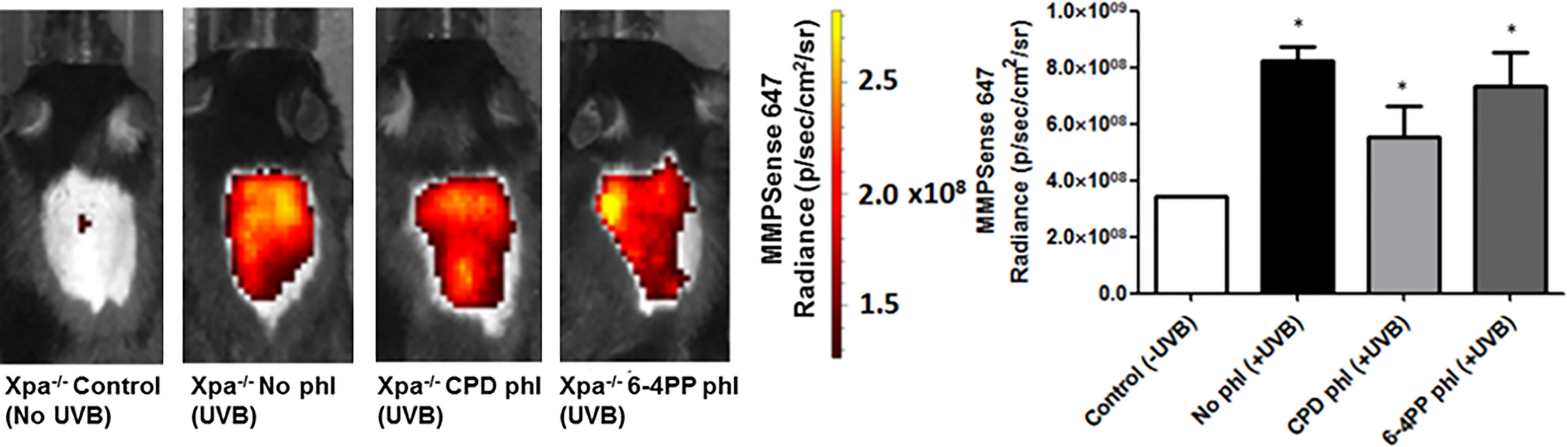
*In vivo* effects of keratinocyte-specific photorepair of CPDs or 6-4PPs in Xpa-/- mice after a single, high UVB irradiation dose. MMPsense was used to measure in vivo the presence of MMPs in mice skin 24 h after UVB irradiation, n=2. Radiance quantifications of fluorescent and chemiluminescent probes were performed in the central region of the mice exposed dorsal skin with equivalent sized regions of interest using Living Image 4.0 software. Asterisks (*) indicate a statistically significant difference between the designated group and negative, Xpa-/- non-irradiated, controls.

Matrix Metalloproteinases (MMPs), enzymes that modulate innate immunity, and tissue remodeling were measured *in vivo* using a commercial fluorescent probe capable of detecting active members of the MMPs, including MMP2, 3, 7, 9, 12, and 13. Surprisingly, unlike our previous results, the removal of neither photolesion significantly reduced the UVB-induced MMP tissue presence 24 h after radiation (n=2). Although there was a trend towards lower MMP levels after CPD photorepair, it was not significant (p=0.1488) (**Figure 4**).

## Discussion

In this work, we used NER-deficient, *Xpa^-/-^* mice to investigate the *in vivo* effects of transgenic photolyase-mediated lesion-specific removal of CPDs or 6-4PPs in basal keratinocytes after UV radiation. We observed that removal of CPDs strongly prevented UVB-induced hyperplasia and cell proliferation, while the removal of 6-4PP reduced these effects to a lesser extent. In cultured cells, UVB-induced photolesions suppress cell proliferation by stalling cell cycle progression through p53-dependent mechanisms (23, 24). *In vivo*, however, basal keratinocytes display a different response to photolesions, as previously reported using NER-proficient mice expressing CPD-photolyase (17, 19). CPDs not only upregulate the expression of genes typically associated with DNA damage response and pro-apoptotic genes, but also genes associated with cell proliferation (19).

Photorepair of 6-4PPs, however, does not prevent any UVB-induced cell proliferation in NER-proficient mice, an effect interpreted to be due to the rapid repair of 6-4PP lesions by NER (19, 25). Since CPD lesions are generated at a higher rate than 6-4PPs when DNA is exposed to UV radiation (25, 26), it is expected that, in a NER-deficient background, CPDs will be present in higher amounts than 6-4PP lesions. We thus hypothesize that the remaining 6-4PP lesions in *Xpa^-/-^* mice expressing CPD-photolyase do not exceed the necessary damage threshold to initiate cell proliferation signaling in the present study settings. Interestingly, photorepair of 6-4PPs in *Xpa^-/-^* mice also attenuated this response, further indicating that the presence of UV-induced DNA photolesions are a major factor to UV-induced cell proliferation and hyperplasia.

Furthermore, both unrepaired CPD and 6-4PP lesions also contribute to UVB-induced apoptosis in *Xpa^-/-^* mice, as the expression of either photolyase leads to a reduction in this cell death process. These results and previous *in vivo* studies using NER-proficient mice expressing CPD- or 6-4PP- photolyase (17, 19) corroborate with *in vitro* studies (2). The removal of CPD, but not 6-4PP lesions in NER-proficient cells, reduces apoptosis induction, while in XP-A NER-deficient cells, the removal of either lesion resulted in the reduction of apoptosis (2). As previously mentioned, CPD lesions are generated at a higher rate than 6-4PPs as a result of UVB irradiation (25, 26). Therefore, it stands to reason that activation of apoptotic pathways by UV irradiation depends not only on the number but also on the type of photolesion (2).

While less numerous, 6-4PP lesions cause a more pronounced distortion on the DNA molecule, i.e. generating a 44° bend of the DNA helix, contrasting to a 9° helix bend caused by CPDs (3, 27). These structural differences have a significant impact on DNA replication, which is obstructed by 6-4PP, but not CPD lesions (27). In addition, both CPD and 6-4PP lesions stall transcription by RNA polymerases in a cell-free transcription elongation system (28). In cells, RNA pol II has been shown to bypass certain DNA lesions, including CPDs, by a translesion transcription mechanism, albeit with low efficiency (29). Both replication and transcriptional stress can activate proapoptotic signaling (3, 30), and the greater distortion of the DNA molecule by 6-4PP lesions may generate different responses to polymerases (27), explaining the distinct role these lesions have in the apoptotic cell death observed in UVB-irradiated *Xpa^-/-^* mice and in XP-A cells. Interestingly, photolesions have also been implicated in skin inflammation (14, 16, 19), as DNA damage-induced replication and transcriptional stress induces pro-inflammatory cytokines (21, 31–33).

UV radiation promotes inflammation through several distinct mechanisms, such as activation of the inflammasome and pro-inflammatory cytokines, as well as major inflammation-related signaling pathways such as NF-κB and p38 (34, 35). IL-1α has also been suggested to act as a DNA damage sensor, as it colocalizes with CPD lesions and is secreted after genotoxic stress. Interestingly, removal of CPD lesions in NER-proficient animals reduces the pro-inflammatory effects of UV (16, 19), again linking CPD photolesions to inflammation. Our study further characterizes this association by showing that both CPDs and 6-4PPs have a role in these effects on NER-deficient mice, as removing either lesion in basal keratinocytes caused a reduction in UVB-induced neutrophil infiltration and activation in the skin. Similar to the apoptotic response, this suggests that the inflammatory responses elicited by photolesions might also depend on the lesion type, and not only on the amount of damage. Furthermore, these results indicate that basal keratinocytes have a major role in regulating UV radiation-related inflammation, in agreement with previous studies (12, 36).

Despite the anti-inflammatory effect of photorepair, the removal of neither CPD nor 6-4PP displayed any significant effects regarding the level of Matrix Metalloproteinases (MMPs) in the skin following UVB irradiation. Notably, the methodology of the present study did not differentiate between different kinds of MMPs, as the probe used can be activated by MMP 2, 3, 7, 9, 12, and 13. Different MMPs generally have distinct effects and may participate in both inflammation initiation and resolution (37). For instance, MMP2, MMP3, and MMP9 have a role in activating the pro-inflammatory cytokines TNF-α and IL-1β, while MMP3 may also participate in the degradation of mature IL-1β depending on the context (38). Moreover, as the photolyases were expressed only in basal keratinocytes, it is possible that other cells, such as fibroblasts residing in the upper dermis layer (19), also contributed to the release of MMPs. There is also the possibility that photolyase-expressing mice presented a decrease in MMP expression, but no signal reduction could be detected due to an over-saturation of the MMP fluorescent signal caused by a high dose of UVB irradiation on *Xpa^-/-^* mice. Therefore, further studies regarding specific MMPs are required to better elucidate the role of CPDs and 6-4PPs on the induction of these molecules.

In summary, by using *Xpa^-/-^*, keratinocyte-specific photolyase-expressing mice, we were able to demonstrate that both CPD and 6-4PP lesions participate in UV-related effects such as hyperplasia, cell proliferation, inflammation, and apoptosis using *in vivo* NER-deficient models, with keratinocytes having a major role regarding these effects. These results corroborate previous studies concerning photolesion effects on apoptosis and hyperplasia and have novel implications regarding DNA damage as a pro-inflammatory stimulus. These discoveries also have important implications for XP patients, incapable of repairing UV-induced photolesions. These patients have a much higher skin carcinogenesis predisposition and different mutation spectra in skin tumors (39). This could be related the pro-inflammatory effects of both photolesions, with inflammation being a critical factor in tumor progression and DNA damage due to releasing oxidizing agents (40). Furthermore, unlike NER-proficient models, in which CPD lesions are the main photolesion responsible for triggering the studied effects, NER-deficient models have both CPDs and 6-4PPs participating in these effects, with 6-4PPs possibly having a different role in XP tumorigenesis (2), with the tumors from these individuals having different causative lesions compared to the rest of the population. Additional investigations on the molecular mechanisms of the activation of the UVB effects in NER-deficient models could shed light on XP carcinogenesis and how the photolesions interact with the multitude of molecular pathways involved in these UVB-induced responses.

## Data Availability Statement

The raw data supporting the conclusions of this article will be made available by the authors, without undue reservation.

## Ethics Statement

The animal study was reviewed and approved by Ethical committee for experimentation with animals of the Institute of Biomedical Sciences of the University of Sao Paulo (Protocol #121-11-03).

## Author Contributions

GK, CQ, CM conceived the study. CM and CQ. CQ supervised the work. GK,CQ and CM designed the experiments. GK, CQ, CG, WF and JdS performed the experiments. GK, WF and CM analyzed the data. GK prepared the figures and wrote the manuscript with input from CQ, CG, GH, JH and CM. All authors contributed to the article and approved the submitted version.

## Funding

This work was supported by FAPESP (Sao Paulo Research Foundation, SP, Brazil, grants #2019/19435-3 and #2013/08028-1) under the International Collaboration Research from FAPESP and The Netherlands Organization for Scientific Research (NWO, The Netherlands). FAPESP also provided a PhD scholarship and financial support for GSK (#2013/13720-1 and #2015/20368-8). This Project was further supported by the Conselho Nacional de Desenvolvimento Científico e Tecnológico (CNPq, Brasília, DF, Brazil, grant #308868/2018-8), and Coordenação de Aperfeiçoamento de Pessoal de Nível Superior (CAPES, Brasília, DF, Brazil, Financial code 001, grant #88887.337792/2019-00). JHJH acknowledges financial support from the National Institute of Health (NIH)/National Institute of Ageing (NIA) (P01 AG017242; DNA repair, mutations and cell aging), European Research Council Advanced Grant Dam2Age, the European commission EU ITN Address (GA-316390), Dutch research organization ZonMW Memorabel project ID 733050810, ONCODE (Dutch Cancer Society), Deutsche Forschungsgemeinschaft (DFG, German Research Foundation) - Project-ID 73111208- SFB 829 as well as European Joint Project on Rare Diseases, RD20-113, acronym TC-NER. The funders had no role in study design, data collection and analysis, decision to publish, or preparation of the manuscript. The content is solely the responsibility of the authors and does not necessarily represent the official views of the National Institutes of Health.

## Conflict of Interest

The authors declare that the research was conducted in the absence of any commercial or financial relationships that could be construed as a potential conflict of interest.

## Publisher’s Note

All claims expressed in this article are solely those of the authors and do not necessarily represent those of their affiliated organizations, or those of the publisher, the editors and the reviewers. Any product that may be evaluated in this article, or claim that may be made by its manufacturer, is not guaranteed or endorsed by the publisher.
