# Supplementary material for: Photorepair of Either CPD or 6-4PP DNA Lesions in Basal Keratinocytes Attenuates Ultraviolet-Induced Skin Effects in Nucleotide Excision Repair Deficient Mice
Source: Front Immunol. 2022 Mar 29;13:800606. doi: 10.3389/fimmu.2022.800606 (PMC9004445; doi:10.3389/fimmu.2022.800606)
Supplement: Supplementary file 1 [file DataSheet_1.pdf]

Photorepair of either CPD or 6-4PP DNA lesions in basal keratinocytes attenuates ultraviolet-induced skin effects in Nucleotide Excision Repair deficient mice

Gustavo Satoru Kajitani, Carolina Quayle, Camila Carrião Machado Garcia, Wesley Luzetti Fotoran, Juliana Fernandes Rossi dos Santos, Gijsbertus TJ van der Horst, Jan HJ Hoeijmakers, Carlos Frederico Martins Menck

Supplementary Material:

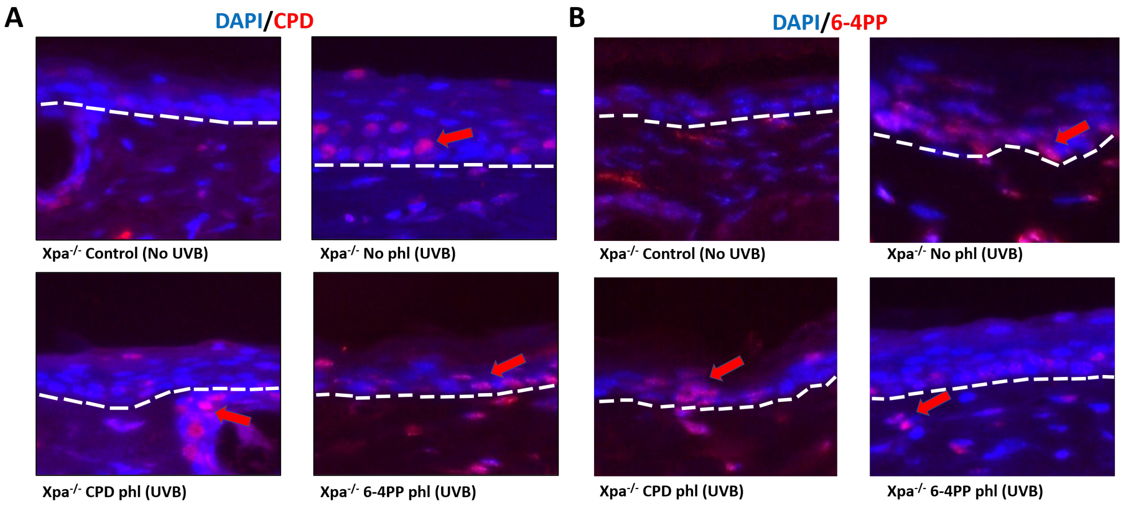

Supplementary Figure S1: Photolyase-mediated repair of UVB induced photolesions in Xpa-/- mice keratinocytes. Immunohistochemistry of CPD or 6-4PP in dorsal skin sections of UVB irradiated (200 J/m^2) and non-irradiated mice, followed by photoreactivation by CPD- or 6-4PP- photolyase (phl). (A) Representative images (100x) of Anti-CPD (Red) merged with DAPI (Blue). (B) Representative image (100x) of Anti-6-4PP (Red) merged with DAPI (Blue). White dotted line designates the basal lamina that separates the epidermal and dermal layers. Red arrows indicate nuclei with photolesion staining. One can observe that the specific lesions are removed depending on the photolyase, and only on the keratinocytes.

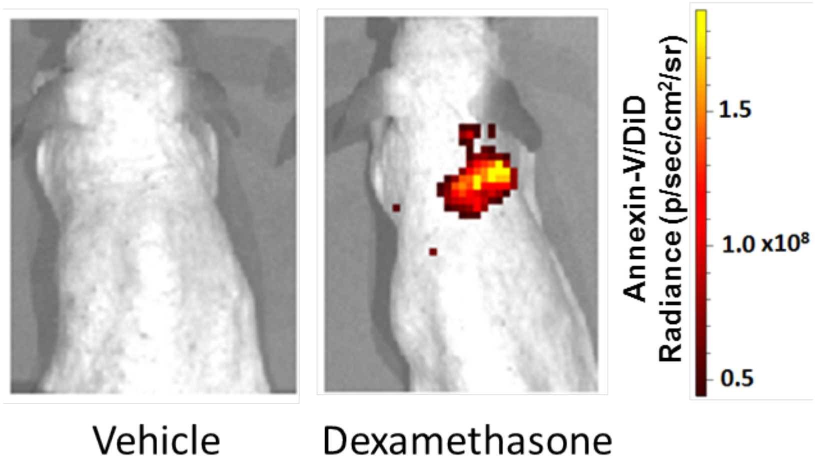

Supplementary Figure S2: Apoptosis detecting nanoparticles validation. Hairless mice received 100 μL of 10 μM mg dexamethasone, a potent inducer of apoptosis in the thymus, injected intraperitonially and imaged after 18 h, with Annexin-V/DiD nanoparticles injected intravenously 3 h prior to imaging.

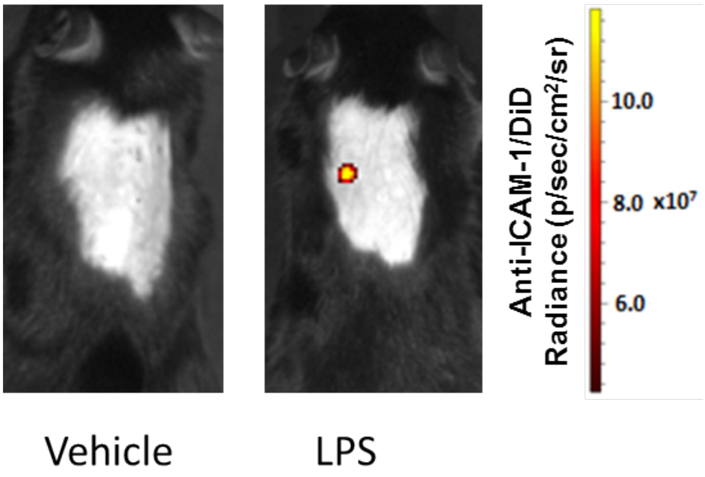

Supplementary Figure S3: Validation of ICAM-1 binding nanoparticle. Black6J mice were injected subcutaneously with 100 μL of 10 mg/ml LPS in shaved dorsal skin and had in vivo imaging performed 6 h following irradiation, with anti-ICAM1/DiD nanoparticles injected 3 h prior to imaging.

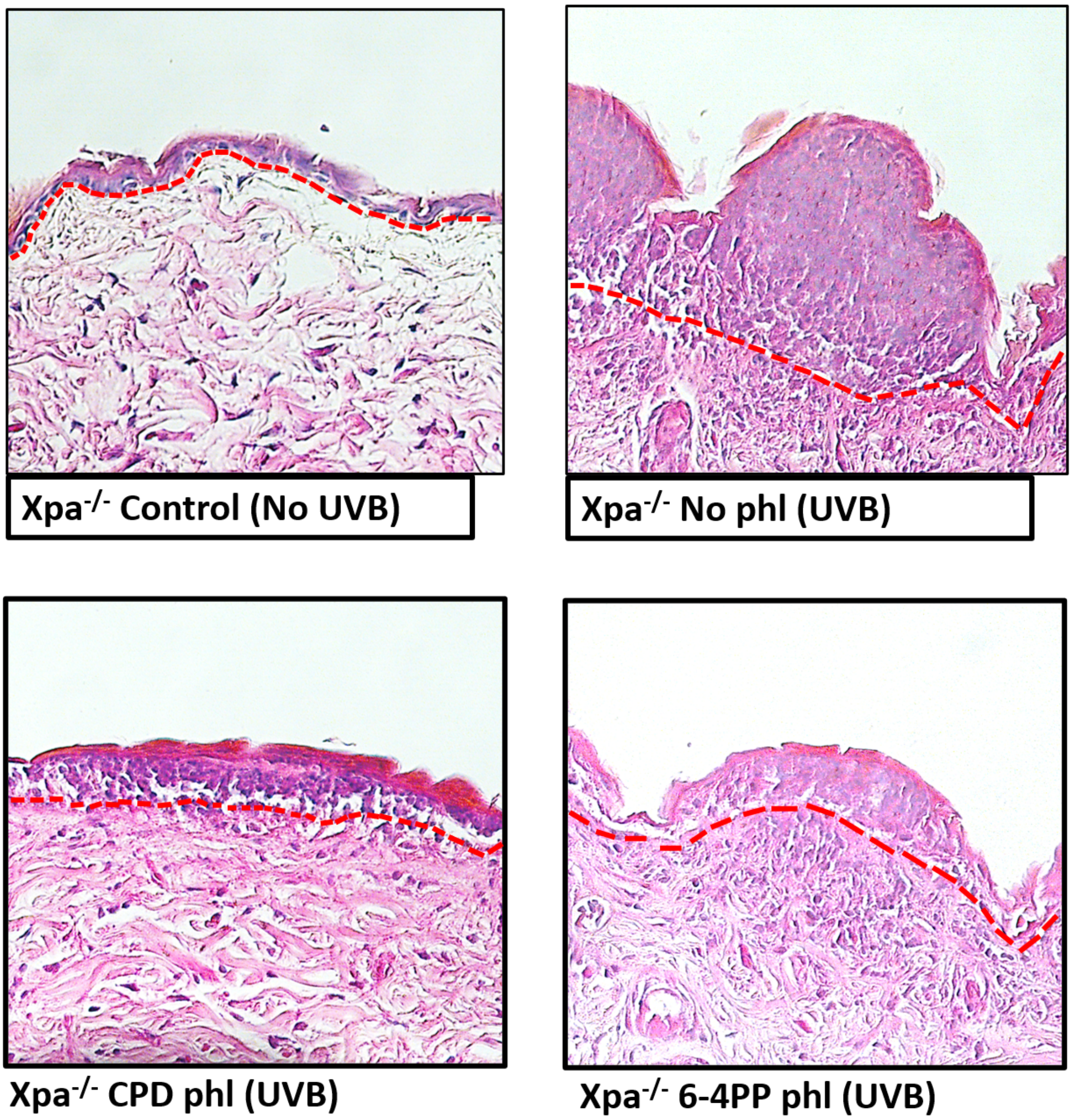

Supplementary Figure S4: Photorepair of either CPD or 6-4PP lesions attenuates leukocyte infiltration in Xpa$^{-/-}$ mouse skin after UVB irradiation. Dorsal skin of *Xpa$^{-/-}$* mice irradiated or not with UVB (200 J/m^2) followed by photoreactivation of CPD- or 6-4PP- photolyase (phl). Tissue was collected 24 h after UVB irradiation, and skin sections were stained with H&E (n = 3). Red dotted line indicates the basal lamina that separates the epidermal and dermal layers. Representative images (40x) displaying leukocyte infiltration in the epidermis of UVB irradiated tissues, and this effect is reduced when photoreactivation occurs.

Supplementary Table S1: Genotyping PCR reagents concentration for CPD and 6-4PP photolyases, *hairless* and *xpa* genes.

Target gene	Primers (pM)	DNTPs (pM)	Buffer 10x (μL)	Taq Polymerase (U)	H_2O (μL)
Photolyases & *hairless*	0.25	0.25	2.25	0.5	q.s. 20
xpa	0.8	0.2	3.75	0.625	q.s. 25

Supplementary Table S2: Temperature cycles used for genotyping PCRs.

Target gene	Steps												
	1		2		3		4		5	6		7	
	T (°C)	t (s)	T (°C)	t (s)	T (°C)	t (s)	T (°C)	t (s)	Repetitions of steps (2-4)	T (°C)	t (s)	T (°C)	t (s)
Photolyases & *hairless*	95	60	95	30	58	30	72	30	30	72	600	4	∞
xpa	95	120	95	30	62	30	72	60	35	72	300	4	∞

Supplementary Table S3: Primer sequences for genotyping PCRs.

Target gene	Primer	5'->3' Sequence
CPD photolyase	CPD P1	TGAGACTCATCTCCCAGGAC
	CPD P2	CACCAATGCCATGTGTTTGC
6-4PP photolyase	6-4PP P1	GCACGATTCAGCAAGCAAGG
	6-4PP P2	CGGTACCTCTACCTATTTGAGTT
hairless	HR P1	GCGTTACTGCAGCTAGCTTG
	HR P2	TGTAGCCTGTGGTCGCATAG
	HR P3	CTCCTGTTTGCTTGGTCATC
xpa	XPA-PGK2 154	GGCCACTTGTGTAGCGCCAA
	XPA26 155	GTGTCAGGCATAAGATCTATGACAA
	XP47 156	AGGCAAGCACCTGCAGCTGT
